# School-based self-management intervention using theatre to improve asthma control in adolescents: a pilot cluster-randomised controlled trial

**DOI:** 10.1186/s40814-022-01031-1

**Published:** 2022-03-23

**Authors:** Katherine Harris, Chris Newby, Gioia Mosler, Liz Steed, Chris Griffiths, Jonathan Grigg

**Affiliations:** 1 Centre for Genomics and Child Health, the Blizard Institute, Queen Mary University of London, London, UK; 2 Centre for Primary Care and Public Health, Yvonne Carter Building, Queen Mary University of London, London, UK

**Keywords:** Paediatrics, Asthma, Self-management, School

## Abstract

**Background:**

Children with poorly controlled asthma have higher rates of unplanned healthcare use and school absences, as well as lower rates of medication adherence and knowledge. They also feel less comfortable using their medication at school, due to social fears and bullying. In this study, this was addressed through two school-based self-management interventions piloted to determine which one to use in a full trial.

**Methods:**

We sought to assess the feasibility and acceptability of two school-based self-management intervention aimed at improving asthma control. Schools in London were randomised to (i) a theatre workshop for the whole year group aimed at raising awareness of asthma in schools, followed by self-management workshops for children (full intervention), (ii) theatre workshop alone (theatre only), or (iii) usual care (controls). Opt-out consent was obtained from parents. The study was a cluster randomised pilot trial, using London schools as the unit of allocation. Our primary aim was to assess the feasibility of delivering a self-management intervention in schools aimed at improving the asthma control test (ACT) score at 6 months. Secondary outcomes included acceptability of the school-based interventions, suitability of the theatre intervention and the full intervention with the self-management workshops, and generation of randomised data to inform future power calculations. Data were analysed by generalised mixed-effect models.

**Results:**

The recruitment strategy for this trial was effective. Five schools were randomised to full intervention (189 children), four to theatre only (103 children), and six to controls (83 children). Asthma control test (ACT) score at baseline and 6 months was obtained from 178/358 participating children. Compared with the controls, there were no large differences found in ACT score with the full intervention; knowledge and perception of asthma improved though. GP and hospital visits increased in the full intervention group. Compared with controls, ACT score was unchanged in the theatre only group.

**Conclusion:**

The asthma self-management intervention trial in schools is feasible and acceptable. The full intervention consisting of both theatre and self-management workshop for asthmatics tended to be better suited to improve outcomes than the theatre intervention on its own. This full intervention should be the one carried forward into a main trial if funding for further research was sought. Further work is needed to understand why there was evidence that unscheduled visits to healthcare professionals increased with the full intervention.

**Trial registration:**

The study was registered on the clinical trials database on 14th May 2018 (ID NCT03536416).

**Supplementary Information:**

The online version contains supplementary material available at 10.1186/s40814-022-01031-1.

## Key messages

1. What uncertainties existed regarding the feasibility?

Can a self-management and peer support intervention for children with asthma aimed at improving asthma control be delivered in schools?

2) What are the key feasibility findings?

Delivering the intervention in schools was feasible, but more support of schools is needed to prevent early dropout. Although compared with controls knowledge and perception of asthma improved in the full intervention group, neither the full intervention or the theatre intervention alone improved asthma control test score at 6 months.

3) What are the implications of the feasibility findings for the design of the main study?

In this pilot study, we found evidence that a school-based asthma intervention improves knowledge and perception, but that this needs to contain both the theatre component and the self-management workshop. Further work is needed to understand the drivers of poor control before planning a large trial powered on ACT score. A new intracluster correlation coefficient was found in the more suitable pilot population along with other statistics that can be used to further inform a power calculation for a main trial if needed.

## Introduction

In the UK, 1.1 million children and young people suffer from asthma, making it the most common chronic condition among children. According to the Global Initiative for Asthma [1], the aim of treatment is to achieve good asthma control due to effective management and use of a prescribed preventer medication where necessary. However, in a recent observational school-based survey of 766 children with asthma in London secondary schools, we identified a significant proportion (49%) of asthmatic children with poor asthma control detected by the validated asthma control test (ACT) score of < 19 [2]. We also found a high level of nonadherence with reliever (29%) and preventer (56%) asthma medication, concerns about stigma, and a poor understanding of asthma [3]. In a subsequent systematic review of school-based self-management interventions for children with asthma, we identified schools as a potentially important site for implementing a successful intervention, since the meta-analyses found that school-based interventions for asthma improved rates of unscheduled care and health-related quality of life and medication use [4, 5]. We also performed qualitative comparative analyses which highlighted that a theoretical framework for interventions was associated with increased likelihood of a beneficial effect [4, 5]. Based on previously collected evidence, barriers of self-management behaviours were identified using the COM-B method by Susan Michie [6], which were subsequenly mapped to behavioural targets. Idea finding techniques were employed to develop intervention elements that address each target. This approach resulted in a rich and varied intervention, including educational videos and interactive games as part of the workshop, as well as the theatre element. The theatre element was developed to address issues around medication adherence, as well as to target empowerment to self-manage for children with asthma. The theatre intervention, as well as the intervention development, has been further described in our previous publications [7, 8].

In the present study, we aimed to assess the feasibility of (i) recruitment for a theatre-based intervention in schools, aimed at improving asthma control among children and young people aged 11–13 years via improved self-management behaviours, including better adherence and improved knowledge of asthma, and (ii) generating data on asthma control to power a future study. To achieve this aim, we performed a pilot randomised trial of a school-based asthma intervention with 3 arms: (i) a “full intervention” group, who received both a theatre workshop for the entire year group and self-management workshops for children with asthma; (ii) a “theatre only intervention” group, who received the theatre workshop for the entire year group; and (iii) a “control” group, who received only usual care. The trial’s primary outcome was change in asthma control test score between the full intervention and control groups.

### Aims

The aim of this study was to assess the feasibility of recruiting secondary schools in London to a school-based self-management intervention for children with asthma. We also sought to look at whether a theatre intervention alone for whole year groups was sufficient to improve outcomes for children with asthma or whether a combination of a theatre intervention and self-management workshops for children with asthma was required. The primary outcome measure in this study was asthma control, measured using the asthma control test. A secondary outcome of this study was to measure the suitability of asthma control as a primary outcome. The second secondary outcome was to generate statistics of children with asthma to inform a new power calculation for a two-arm main trial. Finally, the third secondary outcome was to ascertain an estimate of the intracluster correlation coefficient (ICC) for asthma control.

## Materials and methods

The hypothesis underlying this pilot study was that a school-based self-management intervention will show evidence of effectiveness at improving asthma control, through improved medication adherence and increased peer awareness. This study was approved by the Queen Mary Research Ethics Committee (QMERC2017/77) and required both parental opt-out consent and student written assent. The schools provided consent for whole year groups to engage with the theatre performance. This trial is registered on the clinical trials database (registration number: NCT03536416).

This study had four objectives. The first was to test the feasibility of recruiting secondary schools to facilitate the delivery of a full school-based intervention to secondary school children with asthma, aimed at improving asthma control. Asthma control was assessed by ACT scores at 6 months, compared with control groups, adjusting for school effect and baseline ACT scores. The second key objective was to determine which intervention (theatre intervention for whole year groups or a threatre intervention followed by self-management workshops for children with asthma) would be carried forward to a main trial in terms of proof of concept.

The third objective was to ascertain an estimate of the intracluster correlation coefficient (ICC) for asthma control. The fourth objective was to measure the feasibility of collecting data on secondary outcomes, including medication adherence, unscheduled care, asthma attitudes and perceptions, school attendance, knowledge of asthma, and beliefs about asthma medication. The development of the intervention was informed by our previous systematic review [4, 5] and school-based study [3].

### Changes from the published protocol

This is a pilot study to ascertain whether a theatre intervention alone is sufficient to improve outcomes for children with asthma, through raising awareness of asthma in school.

### Trial feasibility — assessment

The feasibility of the trial was measured through recruitment and retention of secondary schools and completion rates of the questionnaire by participating students.

#### Objective one: feasibility and acceptability — recruitment

Secondary schools in London were approached by the research team. All schools in London were invited to participate initially by email, then by telephone calls and targeted emails to a named teacher, where available. Participating children were required to be attending year seven or eight (11 to 13 years of age). The theatre workshop was delivered to all children in these year groups in both the full intervention and the theatre only intervention groups. In the full theatre and self-management workshop intervention, only participants with doctor-diagnosed asthma were eligible for asthma workshops.

**Fig. 1:**
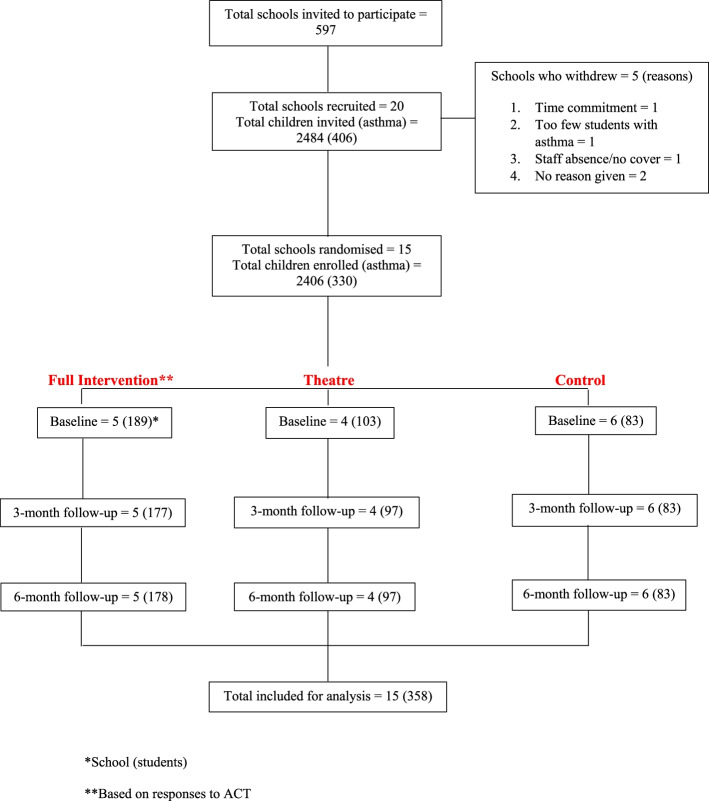
CONSORT diagram. *School (students). **Based on responses to ACT

Acceptability and reasons for refusal were logged and are reported in the CONSORT diagram in Fig. 1. Intervention success was defined as a school retention rate of 70% or higher at 6-month follow-up. This study included three data collection sessions, including half a day of self-management workshops for children in the full intervention group. Acceptability was measured by retention throughout the study.

#### Primary objective two: trial feasibility — measuring asthma control

Data were collected from all children reporting doctor-diagnosed asthma in each class. Data were obtained by questionnaire at baseline (pre-intervention), and at 3, 6-, and 12-month post-intervention (Additional file 1) and included the five-item Asthma Control Test (ACT) [9].

### Secondary patient-centred outcomes

Questionnaire data was collected on the measure medication adherence using the 11-item Medication Adherence Rating Scale (MARS) [10], self-reported healthcare use due to asthma, self-reported school attendance, beliefs about asthma medicines using the 10-item Beliefs about Medicines Questionnaire (BMQ) [11], perceptions about asthma, using the nine-item Brief-Illness Perception Questionnaire (B-IPQ) [12], and questions about asthma knowledge and general healthcare use. Other sections were the same as that used in our previous school-based observational study [3].

### Randomisation

Schools were randomly allocated to one of the three intervention groups using an online computer program (https://www.randomlists.com/team-generator) [8]: the control group received usual care; the intervention group 1 (the “full intervention” group), received the theatre workshop, plus four additional self-management workshops for children with asthma only; and intervention group 2 (the “theatre only” group), who received a theatre workshop to all students in years seven and eight. Full details are reported in the published protocol [13].

### Control group

The schools in the control group received usual care for the duration of the intervention. Usual care included no external input from the reviewers, and the students continued to manage their asthma according to their current plan. At the end of the study, once all the follow-up data had been collected, each control school was offered the theatre workshop.

### Theatre intervention group

The theatre intervention for the “full intervention” and “theatre only” schools was developed and delivered in collaboration with a theatre company [14], with expertise in addressing sensitive subjects in schools through theatre. Theatre workshops delivered the “In Control” play which was written with the aim of facilitating peer support through increasing awareness and understanding of asthma. The theatre performance was delivered to all students in the “full” (theatre and self-management workshop) and “theatre only” intervention groups. The theatre workshop was delivered in schools over a period of 2 h. The first hour included the theatre performance, followed by an hour discussion, facilitated by the actors. The play was set in a secondary school and followed three students in detention. One of the students was considered a “cool kid”, another was her friend, and a third was a colleague in their class. The performance was set across five school days, and during each day, the main character began to experience asthma symptoms that became gradually more severe as she tried to hide it from her peers. By the final day, the main character had an asthma attack, and her friend was able to help manage her attack by administering her reliever inhaler. At the end of the play, the main characters remained in role and engaged in a discussion with the audience of school children about the play and the behaviour of the characters in it. Details about the development of the play are previously reported in a documentary video called “In Control”, found here: https://www.youtube.com/watch?v=1vYhnlWpMUw.

### Full intervention group

In the full intervention group, exposure to the theatre play and group discussion was followed by four self-management workshops limited to those children with doctor-diagnosed asthma. The development of the theoretically informed intervention has been previously reported [7]. In brief, workshops included interactive elements, such as role play, videos, and quizzes to teach children about asthma, including the role of different medications, effective doctor-patient communication, and how to deal with an asthma exacerbation [15]. Each workshop lasted approximately 1 h and was delivered consecutively, on 1 day. The self-management workshops included a video, developed in collaboration with Parkour, found here: https://www.youtube.com/watch?v=VlavDQPmsC8.

### Theoretical framework

The intervention was developed in accordance with the UK Medical Research Council guidance for developing complex interventions [16]. The PRECEDE-PROCEED model [17] and behaviour change wheel [6] were also used as frameworks for developing the intervention. The main behaviour was improved self-management, which was divided into six sub-behaviours. Barriers to each sub-behaviour were mapped according to the COM-B framework by S. Michie [15]. These barriers to improved self-management of asthma were translated into behavioural targets, such as “understanding about different triggers”. The COM-B and theoretical domain elements of the behaviour change wheel [15] were applied to translate identified behaviours into specific interventions, by targeting each behaviour with behaviour change techniques. More information about how the intervention was developed and the theory used to underpin the intervention can be found in our intervention development publication [7].

### Sample size

We calculated that a minimum of 360 children were required for this study from 18 schools, originally 9 in control and 9 in intervention, as part of the feasibility was which intervention to use. This would be achieved by recruiting 6 schools to each arm of the intervention, with at least 20 students with asthma from each school. A 15% attrition rate was used to allow for a more conservative estimate of the number of children and schools required to achieve a significant change in our primary outcome. To account for dropout, we planned to recruit 20 schools.

The study design was a cluster randomised trial, with randomisation by school using an intracluster correlation coefficient (ICC) of 0.05. Although a pilot study, we performed an a priori power calculation to detect a three-point difference in ACT score (minimal important difference [18]) between the intervention and control groups, and with a standard deviation of 4.4 from our previous school-based study [3], creating an effect size of 0.68, a type II error rate of 20% (80% power) was used to reflect the study being a pilot, and a type I error rate was originally 5% but due to the three armed nature of the trial was adjusted to 5/3 = 1.7% for multiple comparisons, This gave us 46.5 in each arm or *N* = 139.5 for the whole trial without adjusting for clustering. Calculating the design effect (DE) and given our *ICC* = 0.05 and we expect 20 children in each cluster, DE = 1+(number in each cluster-1)*ICC = 1.95. Adjusting sample size for the design effect and allowing for 15% attrition within schools give us 139.5 × 1.95 × 100/85, i.e. *N* = 320. We then factor in school attrition that 2 schools might drop out with 20 kids in each bringing our recruitment total to *N* = 360, 18 schools with 20 kids with asthma in each.

And an error rate assumed to be 0% (since the students inputted data directly onto iPads, which were checked after for completion), an attrition rate of 15%, as reported in our protocol [13].

### Statistical analysis

In this pilot study, the primary outcome (ACT score) was compared between the full intervention group and the control group using *t*-tests and generalised mixed-effect models, where appropriate. Analysis was by intention-to-treat and control was dichotomised into a binary variable, including good and poor control. Generalised mixed-effect models were used to understand the random variability from collecting data at multiple timepoints and allowing for the cluster effect of schools. Results are expressed as beta (standard error of the mean (SEM)). The beta score indicates the change from control to comparator intervention, a positive score indicates an increase in intervention compared to control, and a negative score indicates decreases in intervention compared to control. The ACT change score, calculated using the ACT pre- and post-intervention, was used to measure the change in outcomes from baseline to post-intervention.

The significance level in this study was *p* < .05. All statistical analyses were conducted using IBM SPSS statistics (version 23) and GraphPad Prism version 9.

## Results

### Trial feasibility, recruitment, and retention

Of the 597 schools contacted, 20 subsequently agreed to participate. Five schools then dropped out because of concerns about time commitment, and staff shortages and too few students officially registered with asthma (CONSORT diagram, Fig. 1). Since recruitment was limited to the period October 2018 to January 2019, additional schools could not be found to replace schools that dropped out. A total of 375 students from 15 schools with doctor-diagnosed asthma were recruited to the study. No school dropped out at 6 months; however, some students did not complete the questionnaire after baseline (*n* = 17). The total number of children included for analysis was *n* = 358. (11 students in the full intervention group, 6 children in the theatre group, and 0 children in the control group dropped out between baseline and 6 months).

Acceptability was measured by retention throughout the study, which is reported in the CONSORT table in Fig. 1. In total, 17 children dropped out of the study between baseline and 6 months, including 11 in the full intervention group and six in the theatre group. No children were lost to follow-up in the control group. All schools who participated in the data collection at baseline were retained in the study until the end.

### Demographics

The characteristics of the participating schools and students are shown in Table 1. At baseline, the groups were comparable for gender, although there were more males in the study than female students. The control group had a lower proportion of black ethnicity, compared with the “full” and “theatre only” intervention groups, which were comparable. There were no differences between the three groups for ACT score; however, the “full” peer and workshop intervention” group had a lower proportion of children with optimal asthma control (i.e. ACT score > 19), compared with the other groups.

**Table 1:** Characteristics of participating schools and children with asthma at baseline

	**Total** ***N***	**Control** ***N*** **(%)**	**Full Intervention** ***N*** **(%)**	**Theatre** ***N*** **(%)**
**Type of school**				
**Academy**	8	3 (50)	4 (80)	1 (25)
**Comprehensive**	5	3 (50)	1 (20)	1 (25)
**Grammar**	1	0	0	1 (25)
**Independent**	1	0	0	1 (25)
**Baseline asthma records**				
**Students with asthma**	375	83 (22.1)	189 (50.4)	103 (27.5)
**Student demographics**				
	**Total** ***N*** **(%)**	**Control** ***N***	**Intervention** ***N***	**Theatre** ***N***
**Gender**				
**Male**	240 (64)	53	124	63
**Female**	135 (36)	30	65	40
**Age**				
**11**	147 (39.1)	39	69	39
**12**	180 (47.9)	39	98	42
**13**	43 (11.4)	5	18	20
**Missing**	6 (1.6)	0	4	2
**Ethnicity**				
**Black**	88 (23.4)	10	52	26
**East Asian**	9 (2.4)	0	3	6
**South Asian**	113 (30.1)	24	42	47
**White**	84 (22.3)	25	51	8
**Mixed**	60 (16.0)	20	29	11
**Other**	19 (5.1)	4	11	4
**Missing**	2 (0.5)	0	1	1
**Study outcomes** **Asthma control at baseline** ***N*; mean (SEM)**				
	**Total** ***N*** **(%)**	**Control**	**Intervention**	**Theatre**
**ACT score**	337	75; 19 (0.53)	171; 18 (0.34)	91; 20 (0.43)
**Optimal control**	167 (49.6)	41	76	50
**Suboptimal control**	165 (49.0)	34	41	90
**Missing**	5 (1.5)	8	23	12
**Secondary outcomes at baseline per student** ***N*; mean (SEM)**				
	**Total** ***N***	**Control**	**Intervention**	**Theatre**
**Medication adherence**	376	83; 5.54 (0.29)	190; 5.03 (0.18)	103; 5.11 (0.25)
**GP visits**	337	75; 0.52 (0.10)	172; 0.67 (0.08)	90; 0.37 (0.07)
**Hospital visits**	336	75; 0.23 (0.07)	171; 0.39 (0.06)	90; 0.14 (0.43)
**School absences**	336	75; 0.48 (0.11)	171; 0.59 (0.08)	90; 0.31 (0.08)
**Lesson absences**	335	74; 0.28 (0.07)	171; 0.37 (0.06)	90; 0.32 (0.07)
**PE absences**	336	75; 0.56 (0.11)	170; 0.61 (0.07)	91; 0.30 (0.07)
**Necessity score**	323	74; 16.47 (0.49)	159; 14.59 (0.41)	90; 14.30 (0.49)
**Concerns score**	322	75; 12.52 (0.47)	156; 12.79 (0.37)	91; 12.13 (0.45)
**Asthma Perceptions**	300	74; 47.26 (1.00)	142; 47.98 (0.78)	84; 46.38 (0.93)
**Knowledge**	297	76; 6.82 (0.30)	139; 6.90 (0.22)	82; 7.57 (0.29)

### Asthma control

At 6 months, ACT scores increased both groups compared to the control (full intervention (*M* = 1.16 (0.69)) and (theatre group, (*M* = 0.02 (0.67; paired *t*-test; Table 2))).

**Table 2:** Assessment of a school-based intervention on asthma control

Outcome*	N	Mean (SEM) 6 months	95% ***CI***
**Full intervention group ACT change score**	171	0.83 (0.30)	0.25 to 1.42
**Theatre group ACT change score**	91	0.27 (0.28)	−0.29 to 0.84
**Full intervention group ACT score**	83	1.16 (0.69)	−0.21 to 2.52
**Theatre group ACT score**	83	0.02 (0.67)	−1.30 to 1.35

*Compared with control; *ACT* Asthma Control Test, *SEM* standard error of mean, *CI* confidence interval

An estimate of the intracluster correlation coefficient was calculated for the primary outcome at baseline and 6 months. At baseline, the ICC was 0.008 and 0.000 at 6 months, indicating that ACT scores are not affected by school.

A mixed-effect model for the primary outcome showed no effect on asthma control score in both the full intervention (*B* = −0.71 (*SEM* = 0.42)) and theatre groups (*B* = −0.63 (*SEM* = 0.47)) (Table 3).

**Table 3:** Mixed-effect model for asthma control at 6 months

	Control vs full intervention			Control vs theatre only		
	Full ***N***	Beta (SEM)		Theatre ***N***	Beta (SEM)	
Primary outcome Control: ***N*** = 75						
**ACT score**	164	−0.71 (.42)		91	−0.63 (0.47)	

### Other outcomes

Data for remaining ten outcomes are presented in Table 4. Although there was an increase in measures of perceptions of asthma (*M* = 48.33 (1.19)) and knowledge (*M* = 8.36 (0.41)) in the full intervention group compared with controls, this group had more GP and hospital visits (*M* = 0.52 (0.11)) and (*M* = 0.39 (0.10)), respectively, and PE lesson absences (*M* = 0.62 (0.12)) compared with controls. Table 5 shows the mixed-effects model for secondary outcomes.

**Table 4:** Scores for secondary outcomes at 6 months

	Control			Full intervention			Theatre only		
	***N***	Mean (SEM)	95% ***CI***	***N***	Mean (SEM)	95% ***CI***	*N*	Mean (SEM)	95% ***CI***
**Medication adherence**	83	5.94 (0.20)	5.54 to 6.34	191	5.49 (0.15)	5.20 to 5.79	106	5.76 (0.19)	5.38 to 6.13
**GP visits**	83	0.29 (0.06)	0.16 to 0.41	188	0.46 (0.06)	0.35 to 0.57	103	0.29 (0.07)	0.16 to 0.42
**Hospital visits**	83	0.11 (0.04)	0.02 to 0.19	188	0.35 (0.05)	0.24 to 0.45	104	0.15 (0.04)	0.06 to 0.24
**School absences**	83	0.25 (0.07)	0.11 to 0.39	188	0.42 (0.06)	0.31 to 0.54	104	0.25 (0.06)	0.13 to 0.37
**Lesson absences**	83	0.24 (0.06)	0.12 to 0.37	188	0.40 (0.05)	0.30 to 0.50	104	0.38 (0.06)	0.25 to 0.50
**PEaAbsences**	83	0.33 (0.07)	0.19 to 0.46	188	0.57 (0.06)	0.45 to 0.69	104	0.46 (0.07)	0.32 to 0.61
**Necessity**	83	15.58 (0.46)	14.66 to 16.50	186	14.53 (0.35)	13.85 to 15.21	104	14.71 (0.47)	13.78 to 15.64
**Concerns**	83	11.88 (0.45)	10.99 to 12.77	186	12.45 (0.32)	11.82 to 13.08	104	12.04 (0.45)	11.14 to 12.94
**Perceptions**	82	44.40 (0.96)	42.48 to 46.32	183	48.20 (0.68)	46.86 to 49.54	105	47.17 (0.85)	45.49 to 48.86
**Knowledge**	83	7.01 (0.29)	6.43 to 7.59	183	8.47 (0.24)	8.00 to 8.94	105	7.97 (0.27)	7.43 to 8.51

*SEM* standard error of mean, *CI* confidence interval

**Table 5:** Mixed-effect model for effect of the Iitervention on secondary outcomes at 6 months

	Full intervention		Theatre intervention	
	***N***	Mean (SEM)	***N***	Mean (SEM)
**Medication Adherence**	198	5.47 (0.26)	84	5.96 (0.23)
**GP visits**	196	0.52 (0.11)	103	0.32 (0.11)
**Hospital visits**	195	0.39 (0.10)	104	0.15 (0.06)
**School absences**	196	0.48 (0.12)	104	0.27 (0.11)
**Lesson absences**	196	0.44 (0.10)	104	0.38 (0.10)
**PE absences**	196	0.64 (0.12)	104	0.50 (0.11)
**Necessity**	195	14.29 (0.62)	104	14.71 (0.67)
**Concerns**	195	12.26 (0.58)	104	12.04 (0.64)
**Perceptions**	188	48.33 (1.19)	105	47.17 (1.28)
**Knowledge**	187	8.36 (0.41)	105	7.97 (0.40)

*SEM* standard error of mean

## Discussion

This pilot feasibility study found that recruiting large numbers of schools and children with asthma, delivering a school-based self-management intervention, and obtaining information on asthma outcomes are feasible in London secondary schools. Once schools had been enrolled in the study and students had completed the baseline questionnaire, all schools remained involved with the study until the end of the data collection, demonstrating high levels of engagement from schools, which is a key factor in the success of this feasibility study. Analysis of the pilot data found that although the full intervention improved perceptions and knowledge of asthma, no change in asthma control was seen.

Schools were recruited via heads of science teaching staff, who were sent targeted emails informing them of the study. The school staff were then responsible for identifying the students with asthma who may be eligible for the intervention, as well as the whole year groups who would receive the theatre intervention. School staff were unaware which arm of the intervention their school had been allocated to. The randomisation was done basically, randomising schools and not using baseline demographics of schools to stratify the randomisations. This has lead to a possible imbalance in the types of school samples in the intervention and control with the intervention arm having more schools that are culturally diverse and more gender specific and the schools in the theatre arm having worse asthma control at baseline. This imbalance could create confounding, so for the main trial, we would stratify based on the variables average asthma outcomes and type of school so that these school characteristics were evenly distributed between group and intervention schools

Despite the lack of evidence for a beneficial effect on asthma control, evidence that the full intervention improved overall perceptions of asthma is positive, since the 2014 National Review of Asthma Deaths [19] demonstrated that poor perceptions of the risk of adverse outcomes associated with asthma are a contributing factor to mortality among children and young people. But why the invention increased some of the healthcare seeking and school attendance parameters remains unclear. One possible explanation is, since asthma control was no worse in the full intervention group at 6 months, raising awareness of asthma control may have lowered the threshold of children and young people with asthma to seek medical attention, and may have inadvertently given children confidence to opt out of physical education (PE) lessons. Thus, on one hand, this study clearly shows that it is feasible to deliver a self-management intervention in schools; however, more work is needed on understanding the drivers of control and health-seeking behaviours before a definitively powered trial. A key area that should be considered when designing such a trial, is support from healthcare professionals, to ensure that children with poor asthma control are on the appropriate asthma treatment step [20]. Other limitations also exist which should also be addressed in future studies. First, although a large proportion of the planned number of participants were still enrolled at the end of the study, and all schools who engaged at baseline were still engaged at 6 months, schools did drop out at the last moment after enrolment, leading to large discrepancies in sizes between the intervention groups. Feedback from the schools that dropped out before the study began indicated that there was a concern regarding how much time and/or effort would be needed from the teachers for this study. Therefore, a more comprehensive package that includes more staff support (i.e. a funded supply teacher to coordinate the implementation of the study in schools) or a larger financial incentive may be required to keep the schools engaged. Second, children included in this study were not screened for asthma prior to being included, and we were therefore reliant on schools to identify children with asthma from their own records. Although very little data exists on asthma reporting in schools, our earlier observational study [3] highlighted a lower prevalence of asthma in schools than would be expected, based on data from Asthma UK [21]. A third limitation is although our intervention was delivered by trained members of our outreach team, and the theatre workshop was delivered by trained actors working with our partners at Tramshed, whether it is sustainable remains to be determined. To overcome this, suggested changes could include an asthma education program, delivered to whole year groups, and incorporated into the school curriculum as a health education topic. It may also be possible to deliver the theatre performance as a video, and encourage discussion within the classroom, with the teacher acting as a facilitator.

This trial showed that a full intervention, including a theatre workshop for whole year groups to raise awareness of asthma among children without asthma, followed by a series of self-management workshops for children with asthma was more effective at improving outcomes for children with asthma than a theatre workshop alone. This would be taken forward into a main trial, and the primary outcome of asthma control would be considered, alongside asthma education.

In summary, this study shows that it is feasible to deliver a randomised school-based self-management intervention successfully using an opt-out recruitment strategy. This study demonstrated strong engagement from schools, with a high retention rate at 6 months, indicating that this approach has strong validity. But our pilot data do indicate that more work is needed to understand the drivers of asthma control and health-seeking behaviours in this age group. Future research would benefit from assessing the effect of a self-management intervention on the use and maintenance of asthma action plans, as well as the effectiveness of correctly administering medication via short-acting bronchodilators.

## Supplementary Information

**Additional file 1.** Data Collection Questionnaire.

## Data Availability

The datasets used and/or analysed during the current study are available from the corresponding author on reasonable request.

## Abbreviations

ACT: Asthma control test
UK: United Kingdom
GP: General practice
ICC: Intracluster correlation coefficient
MARS: Medication Adherence Rating Scale
BMQ: Beliefs about Medicines Questionnaire
B-IPQ: Brief-Illness Perception Questionnaire
SEM: Standard error of the mean
PE: Physical education
