# Supplementary material for: School-based self-management intervention using theatre to improve asthma control in adolescents: a pilot cluster-randomised controlled trial
Source: Pilot Feasibility Stud. 2022 Mar 23;8:67. doi: 10.1186/s40814-022-01031-1 (PMC8941818; doi:10.1186/s40814-022-01031-1)
Supplement: Supplementary file 1 — Additional file 1. Data Collection Questionnaire. [file 40814_2022_1031_MOESM1_ESM.docx]

**Additional file**

**Additional file 1: Data Collection Questionnaire**

**Personal details**

1. What is your name?
2. Are you male or female?
   1. Male
   2. Female
3. How old are you? _________

1. How would you describe your ethnicity?
   1. White
   2. Black
   3. South Asian (e.g. Indian, Bangladeshi, Pakistani)
   4. East Asian (e.g. Chinese, Japanese, Korean)
   5. Mixed
   6. Other: ____________________________
2. What year group are you in? ________
3. Do you have asthma?
   1. Yes
   2. No (please go to section SIX)
4. If you answered yes to question 6: Do you currently have an Asthma Action Plan?
   1. Yes
   2. No

**Section One: Asthma Control Test**

1. In the past 4 weeks, how much of the time did your asthma keep you from getting as much done at work, school or home?
   1. All of the time
   2. Most of the time
   3. Some of the time
   4. A little of the time
   5. None of the time
2. In the past 4 weeks, how often have you had shortness of breath?
   1. More than once a day
   2. Once a day
   3. 3 to 6 times a week
   4. Once or twice a week
   5. Not at all
3. In the past 4 weeks, how often did your asthma symptoms (wheezing, coughing, chest tightness, shortness of breath) wake you up at night or earlier than usual in the morning?
   1. 4 or more nights a week
   2. 2 to 3 nights a week
   3. Once a week
   4. Once or twice
   5. Not at all
4. In the past 4 weeks, how often have you used your reliever inhaler (usually blue)?
   1. 3 or more times per day
   2. 1 to 2 times per day
   3. 2 to 3 times per week
   4. Once a week or less
   5. Not at all
5. How would you rate your asthma control during the past 4 weeks?
   1. Not controlled at all
   2. Poorly controlled
   3. Somewhat controlled
   4. Well controlled
   5. Completely controlled

***If you had completely controlled asthma in the last 4 weeks***

1. Would you say your asthma has gone away?
   1. Yes
   2. No

**Section Two: Medication Adherence (MARS)**

1. What type of inhaler(s) do you use regularly?
2. Blue inhaler (Salbutamol or Ventolin)
3. Purple inhaler (Fluticasone/Salmeterol or Seretide)
4. Red/White inhaler (Budesonide/Formoterol or Symbicort)
5. Brown/White inhaler (Budesonide or Pulmicort)
6. Brown inhaler (Beclometasone or Becotide)
7. Orange inhaler (Fluticasone or Flixotide)
8. Green inhaler (Salmeterol or Serevent)
9. I don’t take any medication
10. I have other inhalers but I don’t know their names
11. Other …………………………………………………………………………………..
12. Do you ever forget to take your medication?

Yes

No

1. Are you careless at times about taking your medication?

Yes

No

1. When you feel better, do you sometimes stop taking your medicine?

Yes

No

1. Sometimes if you feel worse when you take the medicine, do you stop taking it?

Yes

No

1. I take my medication only when I am sick

Yes

No

1. It is unnatural for my mind and body to be controlled by medication

Yes

No

1. My thoughts are clearer on medication

Yes

No

1. By staying on medication, I can prevent getting sick

Yes

No

1. I feel weird, like a ‘zombie’, on medication

Yes

No

1. Medication makes me feel tired and sluggish

Yes

No

**Section THREE: Healthcare Use**

1. How many times have you had an unplanned visit to your GP/doctor due to your asthma in the last month?
   1. 4 or more times
   2. 2-3 times
   3. 1-2 times
   4. Not at all
2. How many times have you had an unplanned visit to the hospital due to your asthma in the last month?
   1. 4 or more times
   2. 2-3 times
   3. 1-2 times
   4. Not at all

**Section FOUR: School Attendance**

1. How many times have you missed a whole day of school due to your asthma in the last month?
   1. 4 or more times
   2. 2-3 times
   3. 1-2 times
   4. Not at all
2. **How many times have you missed all or part of a regular class or lesson due to your asthma in the last month?**
   1. 4 or more times
   2. 2-3 times
   3. 1-2 times
   4. Not at all

1. How many times have you missed all or part of a P.E. lesson due to your asthma in the last month?
   1. 4 or more times
   2. 2-3 times
   3. 1-2 times
   4. Not at all

**Section FIVE: Beliefs about Medicines**

1. My health at present depends on my asthma medicines

Strongly agree agree uncertain disagree strongly disagree

Having to take asthma medication worries me

Strongly agree agree uncertain disagree strongly disagree

1. My life would be impossible without my asthma medication

Strongly agree agree uncertain disagree strongly disagree

1. Without asthma medication, I would be very ill

Strongly agree agree uncertain disagree strongly disagree

1. I sometimes worry about the long-term effects of my asthma medication

Strongly agree agree uncertain disagree strongly disagree

1. My asthma medication is mystery to me

Strongly agree agree uncertain disagree strongly disagree

1. My health in the future will depend on my asthma medication

Strongly agree agree uncertain disagree strongly disagree

1. My asthma medication can disrupt my life

Strongly agree agree uncertain disagree strongly disagree

1. I sometimes worry about becoming too dependent on my asthma medication

Strongly agree agree uncertain disagree strongly disagree

1. My asthma medication protects me from becoming worse

Strongly agree agree uncertain disagree strongly disagree

**Section SIX: Brief-Illness Perception Questionnaire**

1. How much does asthma affect the lives of people who have it?

0 1 2 3 4 5 6 7 8 9 10

No affect at all Severely affects their life

1. How long do you think people have asthma for?

0 1 2 3 4 5 6 7 8 9 10

A very short time Forever

1. How much control do you think people have over their asthma?

0 1 2 3 4 5 6 7 8 9 10

Absolutely no control Extreme amount of control

1. How much do you think your treatment can help asthma?

0 1 2 3 4 5 6 7 8 9 10

Not at all Extremely helpful

1. Do you think people with asthma experience many symptoms?

0 1 2 3 4 5 6 7 8 9 10

No symptoms at all Many severe symptoms

1. How concerned are you about asthma?

0 1 2 3 4 5 6 7 8 9 10

Not at all concerned Extremely concerned

1. How well do you feel you understand asthma?

0 1 2 3 4 5 6 7 8 9 10

Don’t understand at all Understand very clearly

1. How much does your asthma affect people that have it emotionally (e.g. does it make them angry, scared, upset or depressed)?

0 1 2 3 4 5 6 7 8 9 10

Not at all affected emotionally Extremely affected emotionally

1. How important do you think peer support is for people with asthma?

0 1 2 3 4 5 6 7 8 9 10

Not at all important Extremely important

The next section will ask you a few questions about how much you know about asthma. **Please do not worry, this is not a test and we are not trying to catch you out**.

**Section SEVEN: Asthma Knowledge**

1. Asthma would get worse if people smoke around someone with asthma
2. Agree
3. Disagree
4. Don’t know
5. Preventer inhalers (usually brown) only work if you feel them working immediately
6. Agree
7. Disagree
8. Don’t know
9. Asthma is well controlled if you need to take a blue reliever inhaler once a day
10. Agree
11. Disagree
12. Don’t know
13. Using a spacer with an asthma inhaler makes it easier to get the medicine into the lungs
14. Agree
15. Disagree
16. Don’t know
17. A tight feeling in your chest can be a symptom for asthma
18. Agree
19. Disagree
20. Don’t know
21. You can get addicted to asthma inhalers
22. Agree
23. Disagree
24. Don’t know
25. Both stress and cold air can be triggers for asthma symptoms
26. Agree
27. Disagree
28. Don’t know
29. People with asthma are less likely to get swollen airways
30. Agree
31. Disagree
32. Don’t know
33. You should call an ambulance if a person cannot talk or walk during an asthma attack
34. Agree
35. Disagree
36. Don’t know
37. A spacer should only be used by small children
38. Agree
39. Disagree
40. Don’t know
41. The blue reliever inhaler prevents an asthma attack
42. Agree
43. Disagree
44. Don’t know
45. In an asthma emergency, you can give the blue reliever inhaler 10 puffs in a row
46. Agree
47. Disagree
48. Don’t know
49. People with asthma should take a preventer inhaler (usually brown) when they have an asthma attack
50. Agree
51. Disagree
52. Don’t know
53. Several young people die from asthma every year in the UK
54. Agree
55. Disagree
56. Don’t know

**Section EIGHT: Healthcare Use**

1. How many times have you had an unplanned visit to your GP/doctor in the last month?
   1. 4 or more times
   2. 2-3 times
   3. 1-2 times
   4. Not at all
2. How many times have you had an unplanned visit to the hospital in the last month?
   1. 4 or more times
   2. 2-3 times
   3. 1-2 times
   4. Not at all

***Thank you for filling in our questionnaire*** ☺
